# The Insulin/IGF System in Colorectal Cancer Development and Resistance to Therapy

**DOI:** 10.3389/fonc.2015.00230

**Published:** 2015-10-15

**Authors:** Paolo Giovanni Vigneri, Elena Tirrò, Maria Stella Pennisi, Michele Massimino, Stefania Stella, Chiara Romano, Livia Manzella

**Affiliations:** ^1^Laboratory of Experimental Oncology and Hematology, Department of Clinical and Experimental Medicine, Faculty of Medicine, University of Catania, Catania, Italy

**Keywords:** IGF, IGF receptor, insulin, insulin receptor, colorectal cancer, therapy resistance, insulin/IGF signaling

## Abstract

The insulin/insulin-like growth factor (IGF) system is a major determinant in the pathogenesis and progression of colorectal cancer (CRC). Indeed, several components of this signaling network, including insulin, IGF-1, IGF-2, the IGF-binding proteins, the insulin receptor (IR), the IGF-1 receptor (IGF-1R), and IR substrate proteins 1 and 2 contribute to the transformation of normal colon epithelial cells. Moreover, the insulin/IGF system is also implicated in the development of resistance to both chemotherapeutic drugs and epidermal growth factor receptor targeted agents. The identification of hybrid receptors comprising both the IR and IGF-1R adds further complexity to this signaling network. Thus, a comprehensive understanding of the biological functions performed by each component of the insulin/IGF system is required to design successful drugs for the treatment of CRC patients.

## Introduction

The insulin/insulin-like growth factor (IGF) system is a multifactorial signaling network that modulates energy metabolism, cell growth, and cancer (1).

**Figure 1:**
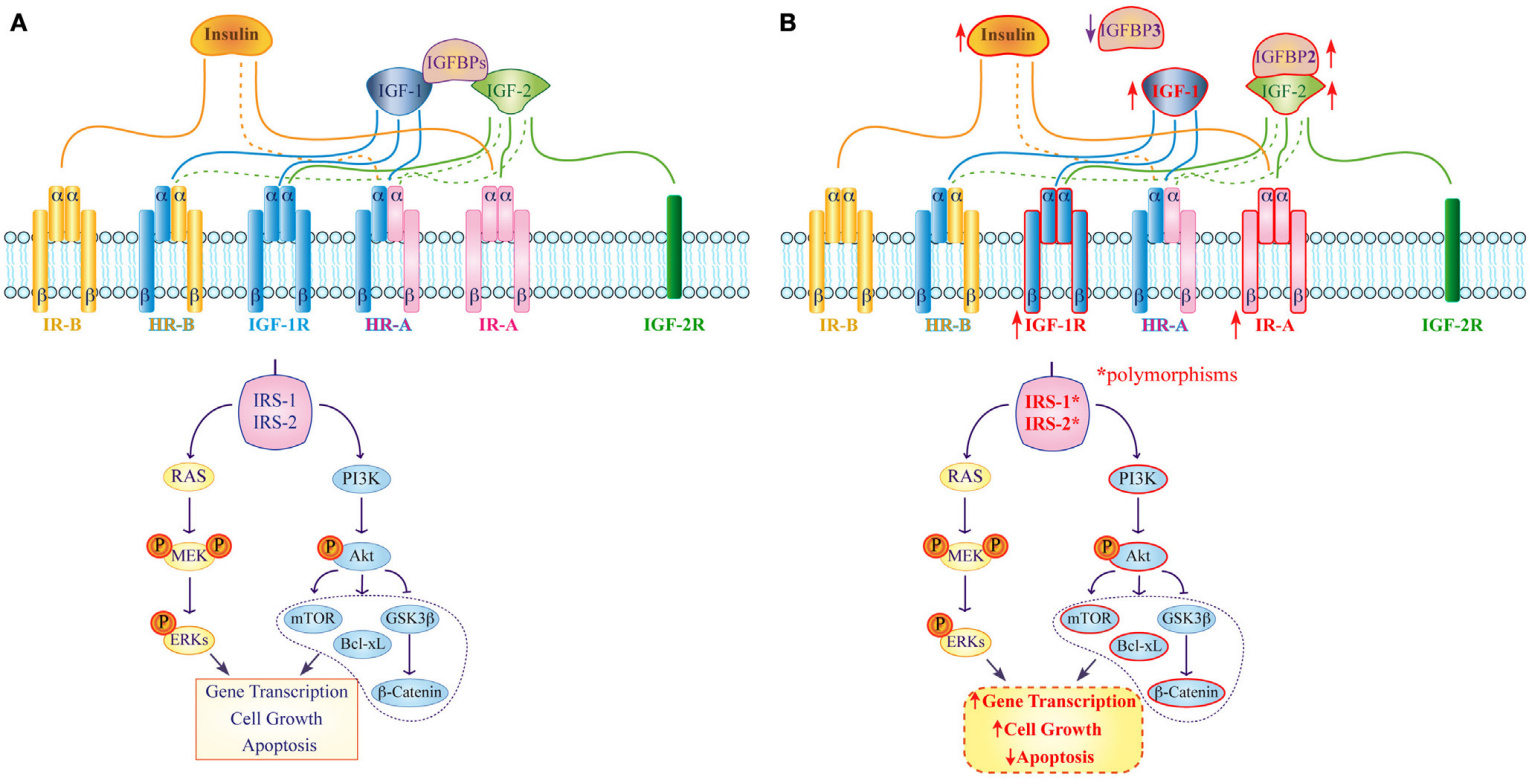
**Expression and function of the insulin/IGF system in normal and colorectal cancer cells**. **(A)** In normal tissue insulin, IGF-1 and IGF-2 bind with high (continuous line) or low (dashed line) affinity to their corresponding receptors and activate specific IRSs, which modulate several pathways involved in gene transcription, cell proliferation, and apoptosis. IGFBPs act as negative regulators that modulate both IGF-1 and IGF-2 functions. **(B)** In colorectal cancer high IR-A and IGF-1R expression (upward arrow) as well as IRS-1 and IRS-2 polymorphisms are involved in up-regulation of the PI3K-Akt pathway. Moreover, IGFPB3 down-regulation (downward arrow) as well as IGFBP2 overexpression (upward arrow) are involved in strengthening IGF-1 functions and in the activation of intracellular signaling pathways that contribute to alter gene transcription, cellular proliferation, differentiation, and apoptosis.

To date, two insulin receptor (IR) isoforms have been described. They differ for the presence of a short exon 11 that can be excised from (IR-A, short isoform) or included in (IR-B, long isoform) the IR coding sequence as a result of alternative splicing (2). Proteolysis of an IR protein precursor generates the mature receptor consisting of two alpha and two beta subunits that form a hetero-tetrameric structure. The alpha subunits display extracellular localization and are responsible for ligand binding. The beta subunits exhibit a trans-membrane segment and an intracellular portion containing the tyrosine kinase domain. While glucose uptake remains the main IR-mediated function, a growing body of evidence suggests that the two IR isoforms have different biological roles with IR-A mostly exerting mitogenic effects and IR-B modulating cell metabolism (3). This hypothesis is supported by the different ligand-binding ability of the isoforms (IR-A recognizes both insulin and IGF-2 with equal affinity, while IR-B primarily binds insulin), and by their diverse tissue distribution (IR-A is more expressed in fetal and cancer tissues, while IR-B is predominant in muscle, liver, and fat) (Figure 1A) (4).

The IGF-1 and IGF-2 receptors (IGF-1R/IGF-2R) show different structures. IGF-1R is homologous to the IR and preferentially binds IGF-1 or IGF-2 over insulin. Otherwise, IGF-2R has a monomeric structure and lacks the catalytic activity. Moreover, this receptor binds only IGF-2, internalizes it, and induces its lysosomal degradation (5). IGF-1R is involved in the regulation of body growth in response to pituitary-released growth hormone (GH). However, activation of IGF-1R can also regulate cell proliferation, survival, and angiogenesis (3, 6, 7). A family of six circulating IGF-binding proteins (IGFBPs) may act as tumor suppressors by limiting IGFs activity (5), although Firth et al. have suggested that IGFBPs may also have IGF-independent effects on cancer growth (8).

IR/IGF-1R hybrid receptors (HRs) have been identified in both immortalized cell lines and human tissues. Specifically, two HRs may be generated by the interaction of the IGF-1R with either IR-A or IR-B. While the intracellular signaling of HRs is not completely understood, it is well known that IR-A/HRs are activated by both insulin and IGFs – although they bind the latter with higher affinity – unlike IR-B/HR that only recognize IGFs (Figure 1A). The current hypothesis is that the preferential association of IR-A or IR-B with the IGF-1R essentially depends on the relative abundance of the two IR isoforms in each tissue (9, 10).

Breast, ovarian, prostate, lung, and colon cancer are the tumor tissues where the insulin/IGF system has been directly linked to tumor development and progression (5, 6). This review will discuss the role of the insulin/IGF system in colorectal cancer (CRC).

## The Insulin/IGF System in Colorectal Cancer

The insulin/IGF system is a critical determinant of CRC development, and also displays important prognostic implications for patients diagnosed with the disease. Indeed, in 2001 Giovannucci and colleagues suggested that multiple markers of hyperinsulinemia (e.g., low physical activity, high body mass index, central adiposity, and high IGF-1 levels) correlate with higher risk of CRC. In their study, CRC incidence was invariably increased after the advent of sedentary lifestyles associated with obesity, and higher availability of processed carbohydrates and saturated fatty acids (11). Moreover, it has been demonstrated that the increased blood levels of insulin in type II diabetes individuals, caused by insulin-resistance, enhance the risk to develop colon cancer (12). In addition, Yang et al. observed further elevated incidence of CRC in diabetic subjects under insulin treatment (13). These results were not surprising as previous evidence had shown that insulin provides mitogenic and pro-angiogenic signals for normal colorectal epithelial cells, possibly increasing their energetic metabolism (14, 15).

An additional study later showed that phosphorylated IR (pIR) is expressed at significantly higher frequencies in low-grade carcinomas, such as in primary specimen from non-small cell lung cancer patients (16), and that higher insulin levels are also evident in patients with dysplastic lesions. Accordingly, Abruzzese and colleagues found high pIR-positive staining during the transition from normal colorectal epithelium to colon adenomas and adenocarcinomas, with strong pIR expression in adenomas and low-grade adenocarcinomas, suggesting that activation of the insulin/IR axis is an early event in colorectal carcinogenesis (Figure 1B) (17).

Signaling downstream of the insulin/IR complex by the insulin receptor-1 and -2 substrates (IRS-1, IRS-2) is also a critical determinant of CRC aggressiveness. Specifically, IRS-1 expression appears inversely correlated to CRC differentiation, supporting a role for IRS-1 in CRC progression and liver metastasis since IRS-1 immunostaining is significantly higher in hepatic metastases relative to both primary CRC and paired colonic epithelium (18). Likewise, IRS-2 mRNA and protein expression positively correlates with normal colorectal epithelium transitioning to adenoma and, subsequently, to adenocarcinoma. Furthermore, IRS-2 overexpression activates the oncogenic PI3 kinase pathway leading to Akt phosphorylation and reduced cell adhesion, both characteristics of invasive CRC cells (Figure 1B) (19). IRS-1 and IRS-2 polymorphisms have also been independently associated with increased CRC risk (20). In fact, the presence of at least one R allele (GR or RR) in IRS-1 position 972 is associated with a higher incidence of CRC. On the contrary, IRS-2 heterozygosity in position 1057 (GD genotype) significantly reduces the risk of developing CRC. Esposito et al. have also analyzed the coding region and short intron-exon borders of both IRS-1 and IRS-2 in 14 CRC cell lines and 33 primary specimens. They identified 21 IRS-1 variants and 18 IRS-2 variants with 7 novel IRS-2 variants, including 4 missenses, 2 in-frame insertion mutations, and 1 silent variant (21). If and how these variants may be involved in the modulation of IRS-2 function in colorectal tumorigenesis remains to be established.

Initial interest for a possible role of IGF-1 in colon cancer development stemmed from observations reporting an increased CRC incidence in patients with acromegaly, a condition characterized by elevated levels of both GH and IGF-1 (22, 23). This potential link was further supported by work from Lahm and colleagues that described frequent IGF-1R overexpression in human colon cancer cells (24). Because it is currently well established that IGF-1 is mostly bound to IGFBP3, the finding that mean IGF-1 levels are higher in CRC patients as compared to normal subjects while IGFBP3 is lower in patients than in healthy individuals strongly suggests a positive association and a negative association of these two proteins with CRC risk (25). These data are also confirmed by several prospective cohort (26, 27) and case-control studies (28, 29). In addition, blood concentrations of IGF-1 have been associated with the risk of prostate and premenopausal breast cancer (30–33), while no significant association of circulating IGF-1/IGFBP3 has been related with ovarian cancer risk (34).

The role of IGF-2 and IGFBP2 in CRC development has been thoroughly investigated by Renehan and colleagues. They examined 92 patients with colon cancer and found mean IGF-2 SD scores (SDS) marginally elevated compared with normal colonoscopy controls. However, mean IGFBP2 SDS were significantly higher in the cancer population compared to controls. Moreover, when considered by disease stage, IGFBP2 SDS significantly increased from early to advanced disease but fell rapidly in patients subjected to curative resection (29). In an additional study, the same group demonstrated that plasma IGFBP2 levels distinguished patients with CRC and advanced colon polyps from healthy subjects. Furthermore, as reported by Liou and colleagues, higher IGFBP2 plasma levels were independently associated with inferior overall survival (OS) of CRC patients (35).

Insulin-like growth factors activate the IGF-1R, which is frequently overexpressed in cancer cells triggering a number of intracellular signaling cascades that enhance cell cycle progression and inhibit apoptosis. Indeed, preliminary findings indicate that IGF-1 and its receptor promote both the growth and malignant transformation of adenomatous polyps (36). In further experiments, a human CRC cell line overexpressing the IGF-1R – HCT116/IGF-1R – resulted in highly invasive tumor and produced distant metastases in murine models, whereas the parental cell line did not. Moreover, IGF-1R overexpression was associated with Akt activation and up-regulation of the anti-apoptotic protein Bcl-xL (Figure 1B) (37). A further study showed that IGF1-R is also involved in the activation of β-catenin in CRC cells. In fact, knockdown of IGF-1R inhibited human CRC cells growth and downstream PI3K and Akt that, in turn, caused the activation of GSK3ß. This protein inhibited β-catenin translocation into the nucleus and the transcription of cell proliferation genes (Figure 1B) (38). Additional data also suggest that both p63 and p73, members of the p53-family, may control colon cancer proliferation via mechanism/s that involve down-regulation of the *IGF-1R* gene. Using transient co-expression assays in colon cancer-derived HCT116, Nahor et al. showed that both proteins inhibit the IGF-1R promoter reducing endogenous IGF-1R levels in a dose-dependent manner. Since mutant p63 and p73 are impaired in their ability to suppress IGF-1R expression, these findings further support a causative role for this receptor in CRC (39). Finally, miR-143 and miR-145 have been recently found to target the 3′ UTR of the IGF-1R. Expectedly, there was an inverse correlation between miR-143/145 levels and IGF-1R expression in CRC. Furthermore, miR-145 also targets the 3′ UTR of IRS-1 and its overexpression dramatically inhibits the growth of colon cancer cells (40, 41).

As for HRs, IR-A/HR is the predominant isoform in most cancer cell lines. In 1999, Frasca et al. reported that IR-A relative abundance was significantly higher in CRC tissues as compared to normal colonic epithelial cells, with median values ranging from 68–73% in cancer to 35–43% in normal tissue (42). By employing the Sox9-EGFP reporter mice, Andreas and colleagues described cell-type-specific differences in IR isoform expression and functions in the intestinal epithelium. They found that IR-A was the predominant isoform in both the undifferentiated intestinal epithelial stem cells and in the rapidly dividing progenitors of the crypt, whereas IR-B expression was increased in post-mitotic entero-endocrine cells and intestinal epithelial cells enriched for other post-mitotic differentiated lineages (43).

## The Insulin/IGF System in Resistance to Colorectal Cancer Therapy

Colorectal cancer is the second leading cause of cancer-related death in the Western population. Almost 25% of patients present metastatic disease at diagnosis, displaying a dismal median OS of 7 months if deprived of appropriate medical treatment (44). However, in recent years, significant improvements in patient survival have been achieved largely due to the emergence of targeted molecular therapies that complement standard chemotherapeutic regimens. The availability of these biologic agents has increased the median OS of metastatic CRC to about 30 months (45). For example, cetuximab and panitumumab, two anti-epidermal growth factor receptor (EGFR) monoclonal antibodies, have been proven efficacious in a subset of patients with metastatic CRC addicted to the EGFR oncogene (46, 47) that do not display mutations in KRAS/NRAS (exons 2–4), BRAF (exon 15), or the catalytic subunit (exon 20) of PIK3 (PI3KCA) (48). Despite these significant advances, the inevitable emergence of drug resistance will ultimately lead to disease progression and, eventually, to patient death.

The activation of insulin/IGF-dependent pathways has been identified as a critical step contributing to several mechanisms of CRC resistance to both conventional and targeted therapeutic agents (49).

**Figure 2:**
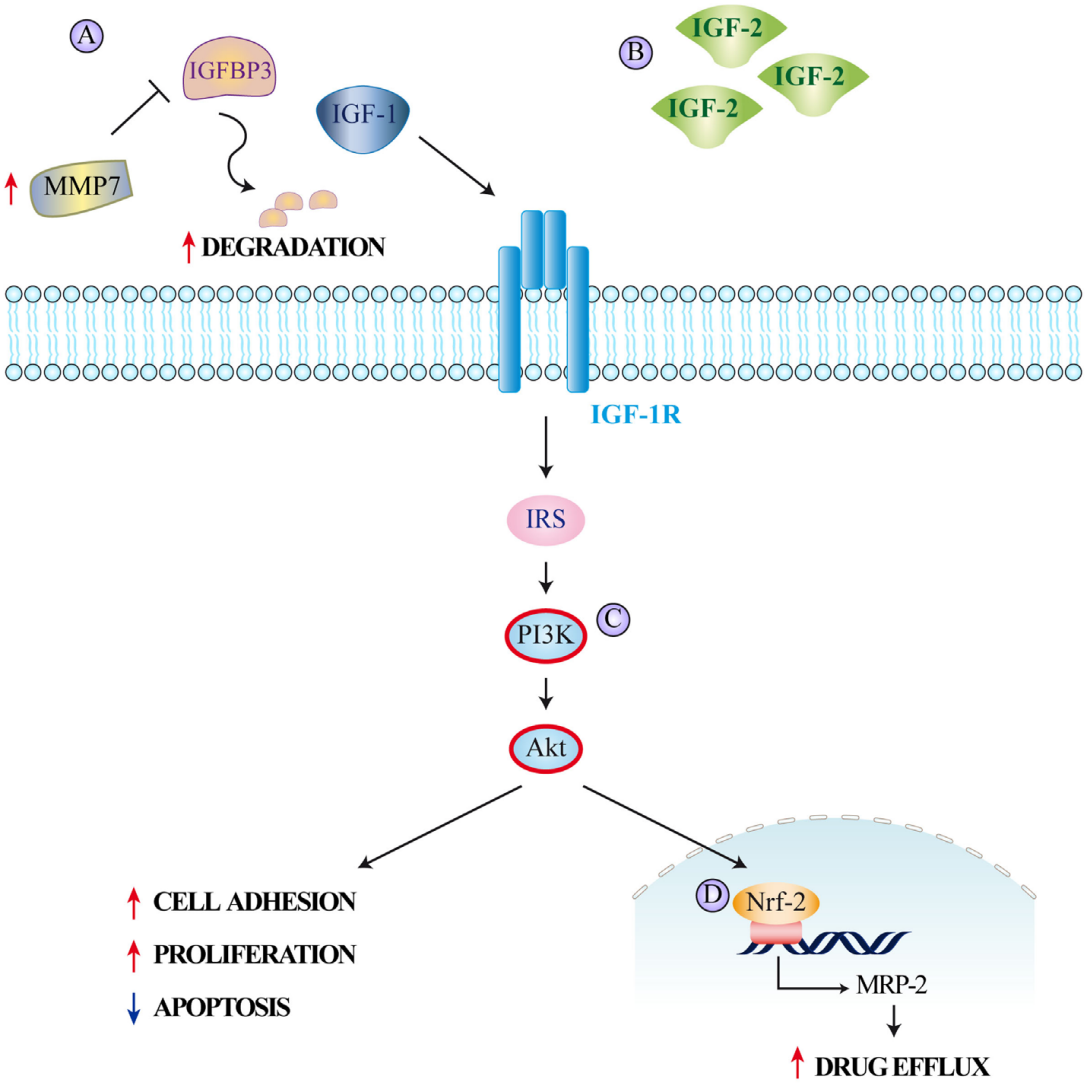
**The insulin/IGF system and colorectal cancer resistance to therapy**. Mechanisms leading to resistance to different therapeutic agents employed in colorectal cancer treatment include **(A)** increase of MMP7 expression causing IGFBP3 proteolytic degradation and subsequent increase in free circulating IGF-1, **(B)** IGF-2 overexpression, **(C)** IGF-1R-mediated PI3K/Akt signaling determining **(D)** nuclear translocation of nuclear factor E2-related factor-2 (Nrf-2) and subsequent induction of MRP-2 expression. The increased drug efflux caused by MRP-2 reduces intracellular drug concentrations.

Scartozzi et al. reported that high IGF-1 expression correlates with poor clinical outcome in wild-type KRAS metastatic CRC patients treated with cetuximab and irinotecan (50). They hypothesized that engaging the IGF-1/IGF-1R system enabled tumor cells to escape anti-EGFR-mediated treatment as a consequence of IGF-1-driven stimulation of the PI3K–Akt pathway. Additional evidence also suggests that IGF-1/IGF-1R polymorphisms are potential predictive/prognostic markers for cetuximab efficacy in metastatic CRC patients presenting wild-type KRAS (51). Another interesting finding is the reported decrease in circulating IGFBP3 levels in CRC patients receiving chemotherapy that develop disease progression (Figure 2A) (52). As Ohashi et al. have previously shown an increase in matrix metallopeptidase 7 (MMP7) in CRC cells unresponsive to chemotherapy, these findings imply that MMP7 could mediate IGFBP3 degradation and enhancement of IGF-dependent cancer cell survival and proliferation (53). Hence, the concurrent increased expression of IGF-1 and MMP7 may be employed as a new marker predicting poor progression-free and OS amongst patients with wild-type KRAS and BRAF (52). IGF-2 is also highly overexpressed in a fraction of CRCs (54) and its activation of the IGF-1R reduces CRC cells response to EGFR inhibition (Figure 2B).

Besides causing overt drug resistance, parallel signals can also induce partial desensitization to treatment. For example, hyperactivation of the IGF-Rs together with HER2 and MET represents another mechanism adopted by CRC cells to escape EGFR oncogene dependency (55–57). Indeed, increased IGF-1R signaling has been often associated with low sensitivity to EGFR blockade (58, 59). An analysis of the functional cross-talk between the IGF-1R and the EGFR has shown that activation of IGF-1R downstream signaling is crucial for the mitogenic and transforming activity of EGFR (60). Both cetuximab and panitumumab inhibit ligand binding to EGFR, thereby suppressing its downstream signaling. Consequently, IGF-1-driven stimulation of the PI3K/Akt pathway provides a rational explanation for at least part of the lack of efficacy observed in a notable fraction of patients with wild-type KRAS tumors treated with EGFR-targeting monoclonal antibodies (Figure 2C). Finally, IGF-1R signaling results in increased expression of the multidrug-resistance-associated protein 2 (MRP-2), which reduces the intracellular concentrations of multiple cytotoxic drugs (61). *In vitro* silencing of the IGF-1R suppresses MRP-2 in CRC cells, thereby increasing the intracellular drug concentration of 5-Fluorouracil, Mitomycin C, Oxaliplatin, and Vincristine. This effect is also mediated by the PI3K/Akt pathway, which causes nuclear translocation of nuclear factor-like 2 and reduces MRP-2 expression (Figure 2D) (62).

Despite promising preclinical evidence, recent trials in metastatic CRC patients with wild-type KRAS have shown no benefit from the combination of anti-EGFR and anti-IGF1-R-directed therapies as compared with EGFR-targeted monotherapy (45, 63). The reasons for these discrepancies are not currently understood and there is still considerable discussion about the validity of IGFs and IGF-1R as viable therapeutic targets in CRC. The identification of predictive biomarkers will be of crucial importance for the further development of innovative therapies based on anti-IGF-1/2 or anti-IGF-1R agents (64).

## Conclusion

The insulin/IGF system plays a pivotal role in the pathogenesis, progression, and prognosis of CRC.

Solid epidemiological and biological evidence indicates that hyperactivation of the insulin/IR pathway represents an early step in colon cancerogenesis, establishing both mitogenic and pro-angiogenic signals that favor neoplastic transformation of normal colorectal epithelial cells. That the insulin/IR axis is actively involved in CRC development is confirmed by the increased invasiveness and inferior outcome of patients displaying elevated IRS-1 and IRS-2 expression.

Likewise, IGFs/IGF-1R signaling is highly active in CRC, contributing to the activation of multiple pathways that increase the aggressiveness of the tumor phenotype. As expression of the *IGF-1R* gene is partially regulated by the p53 paralogs p63 and p73, mutations, deletions, epigenetic silencing, or post-translational inactivation of these tumor suppressors further unleash the oncogenic potential of the IGF-1R in CRC. The inferior prognosis of CRC patients expressing low IGFBP3 or high levels of IGFBP2 suggests that IGFBPs are also implicated in CRC tumorigenesis, either operating as negative regulators of IGFs activity or exerting IGF-independent effects on cancer growth.

The insulin/IGF system also contributes to CRC resistance to both conventional and targeted anti-cancer agents, leading to increased PI3K/Akt signaling that hinders the apoptotic signals triggered by chemotherapeutic drugs and desensitizes CRC cells to the effect of anti-EGFR antibodies. This may at least partially explain the inferior response rates of a subset of metastatic CRC patients presenting wild-type KRAS/NRAS, BRAF, or PI3KCA to these pharmacological agents.

Finally, the detection of IR/IGF-R HRs adds an additional layer of complexity to these intricate signaling networks.

Numerous agents against insulin/IGF system have been tested and even if most of the new compounds performed well in preclinical and early clinical studies, when used in phase II and III trials, they showed disappointing results. Only a detailed understanding of the multifaceted role of the various components of the insulin/IGF system and the validation of predictive biomarkers will allow the appropriate and successful use of anti IGF-1/2 or anti-IGF1R drugs, alone or in combination with other agents, for the treatment of CRC patients.

## Conflict of Interest Statement

The authors declare that the research was conducted in the absence of any commercial or financial relationships that could be construed as a potential conflict of interest.
